# Association between elevated homocysteine levels and obstructive sleep apnea hypopnea syndrome: a systematic review and updated meta-analysis

**DOI:** 10.3389/fendo.2024.1378293

**Published:** 2024-06-03

**Authors:** Jie He, Haiying Zhou, Juan Xiong, Yuanyuan Huang, Na Huang, Jiaqing Jiang

**Affiliations:** ^1^ Clinical Medical College of Chengdu Medical College, Chengdu, Sichuan, China; ^2^ Department of Pulmonary and Critical Care Medicine, The First Affiliated Hospital of Chengdu Medical College, Chengdu, Sichuan, China; ^3^ Department of Rehabilitation, The First Affiliated Hospital of Chengdu Medical College, Chengdu, Sichuan, China; ^4^ Emergency department, The First Affiliated Hospital of Chengdu Medical College, Chengdu, Sichuan, China

**Keywords:** continuous positive airway pressure, homocysteine, meta-analysis, plasma, obstructive sleep apnea-hypopnea syndrome, serum

## Abstract

**Objective:**

This study aimed to distinguish between healthy controls and patients with OSAHS regarding homocysteine (HCY) levels and investigate how individuals with OSAHS respond to continuous positive airway pressure ventilation (CPAP) in terms of serum and plasma HCY levels.

**Methods:**

To ascertain published articles about OSAHS, an exhaustive search was performed across medical databases, encompassing PubMed, Web of Science, EMBASE, CNKI, and Cochrane Library, until January 2, 2024. This study reviewed the literature regarding HCY levels in individuals with OSAHS and control groups, HCY levels under pre- and post-CPAP treatment, the Pearson/Spearman correlation coefficients between HCY levels and apnea-hypopnea index (AHI), and the hazard ratio (HR) of HCY levels concerning the occurrence of major adverse cerebrocardiovascular events (MACCEs) in patients with OSAHS. Meta-analyses were performed using weighted mean difference (WMD), correlation coefficients, and HR as effect variables. The statistical analysis was conducted using the R 4.1.2 and STATA 11.0 software packages.

**Results:**

In total, 33 articles were selected for the final analysis. The OSAHS group exhibited significantly higher serum/plasma HCY levels than the control group (WMD = 4.25 μmol/L, 95% CI: 2.60–5.91, *P*< 0.001), particularly among individuals with moderate and severe OSAHS. Additionally, subgroup analysis using mean age, ethnicity, mean body mass index, and study design type unveiled significantly elevated levels of HCY in the serum/plasma of the OSAHS group compared to the control group. CPAP treatment can significantly decrease serum/plasma HCY levels in patients with OSAHS. Moreover, elevated HCY levels in individuals with OSAHS could be one of the risk factors for MACCEs (adjusted HR = 1.68, 95% CI = 1.10–2.58, *P* = 0.017). AHI scores show a positive correlation with serum/plasma HCY levels.

**Conclusion:**

Patients with OSAHS had elevated serum/plasma HCY levels compared to healthy controls; however, CPAP therapy dramatically decreased HCY levels in patients with OSAHS. In patients with OSAHS, elevated HCY levels were linked with an increased risk of MACCEs, and HCY was positively connected with AHI values. HCY levels may serve as a useful clinical indicator for determining the severity and efficacy of OSAHS treatments.

**Systematic review registration:**

https://www.crd.york.ac.uk/prospero/, identifier CRD42024498806.

## 1 Introduction

Obstructive sleep apnea hypoventilation syndrome (OSAHS) is a prevalent sleep-associated respiratory illness that affects 2%–7% of the middle-aged population worldwide (1). The primary hallmark of OSAHS is repeated hypoventilation or apnea during sleep, which can result in chronic intermittent hypoxia or frequent awakenings (2). Intermittent hypoxia may last for a long time, resulting in severe hypoxemia. These alterations may also cause a variety of cardiovascular problems, including cerebral hemorrhage, stroke, and myocardial infarction (3). However, the underlying mechanisms by which OSAHS leads to cardiovascular disease are not fully understood.

Homocysteine (HCY), which is a cysteine amino acid containing sulfur and is produced by the liver, is capable of engaging in methionine metabolism. A deficit in folate and vitamin B12 inhibits the conversion of HCY to methionine, resulting in hyperhomocysteinemia (4, 5). In recent years, HCY has gained extensive attention. According to epidemiologic data, HCY is strongly correlated with the prognosis of cardiovascular disease and serves as an independent risk factor for the condition (6). Further research is required to determine whether elevated HCY levels are associated with an increased risk of major adverse cerebrocardiovascular events (MACCEs) in individuals with OSAHS. Several studies have demonstrated that serum/plasma HCY levels are greater in OSAHS (7) and associated with severity (8). However, these findings are still contentious. One of the most significant OSAHS therapies is continuous positive airway pressure ventilation (CPAP), which rectifies upper airway collapse during sleep, decreases sleep fragmentation and somnolence, and improves the quality of life (9). Apnea-hypopnea index (AHI) and Epworth Sleepiness Scale were the most commonly reported outcomes used to assess treatment effectiveness (10). Some study reported that HCY levels were also an important indicator for OSAHS severity and progression, especially in elderly patients with cardiovascular diseases (11). However, several studies investigating the effect of CPAP therapy on HCY levels in patients with OSAHS have shown contradictory findings (12).

Therefore, this study used various evidence-based medicine methodologies to perform a more complete meta-analysis of the existing data to compare plasma/serum HCY levels between controls and patients with OSAHS, as well as to determine the effect of CPAP therapy on HCY levels in patients with OSAHS. We also examined whether plasma/serum HCY levels independently predicted MACCEs in patients with OSAHS patients. Finally, we investigated the association between the AHI scores and serum/plasma HCY concentrations in the participants.

## 2 Methods

With the registration number PROSPERO CRD42024498806, this systematic review protocol has been submitted to PROSPERO (https://www.crd.york.ac.uk/PROSPERO/displayrecord.php?RecordID=451081). The present study was conducted following the principles delineated in the Preferred Reporting Items for Systematic Reviews and Meta-Analysis (PRISMA) statement, which mandates the utilization of a systematic evaluation and meta-analysis (13).

### 2.1 Literature retrieval strategy

Non-English and English articles were retrieved from the Excerpt Medica Database (EMBASE), PubMed, Web of Science, China National Knowledge Infrastructure (CNKI), and Cochrane Library databases, respectively. The following terms were used in the retrieval process: upper airway resistance, homocysteine, HCY, continuous positive airway pressure, CPAP, sleep apnea, obstructive sleep apnea, obstructive sleep hypopnea, and sleep-disordered breathing. The article published time spanned from the time of database construction to February 1, 2024. Besides the computerized search, a manual search was performed on all retrieved articles. Articles with potential relevance were evaluated based on predetermined criteria for inclusion and exclusion.

### 2.2 Inclusion and exclusion criteria

(1) All participants underwent polysomnography monitoring; patients with an AHI > 5 were enrolled in the case group, and patients with an AHI< 5 were enrolled in the control group (14). AHI was used to evaluate OSAHS severity (no OSA: AHI< 5, mild OSA: AHI 5–14, moderate OSA: AHI 15–29, severe OSA: AHI≥ 30) (15, 16).
(2) None of the participants were taking any drugs (e.g., methotrexate, folic acid, multivitamins, and others) that could have affected the experiment’s outcome.
(3) No significant statistical differences were found between the OSAHS and control groups about age and body mass index (BMI).
(4) All participants aged > 18 years.
(5) Plasma/serum HCY concentrations were determined using fasting venous blood.
(6) For patients treated with CPAP, the study must have reported HCY concentration values before and after treatment and a course of treatment greater than 1 month.
(7) The study offers ample data for performing a meta-analysis. When the provided information was inadequate for data extraction, the study was excluded.
(8) Abstracts, letters to the editor, animal experiments, and case studies were also excluded.

### 2.3 Data extraction

Three researchers extracted data from each article and standardized it into a common spreadsheet format, which included all pertinent details including the first author’s name, year of publication, type of study design, BMI, sample size, applicable exposures and interventions, duration of treatment, adjusted hazard ratio (HR) and 95% confidence interval (CI) values related to MACCEs, Pearson/Spearman correlation coefficients for HCY concentration, and AHI, among others.

### 2.4 Literature quality assessment

Given that the studies that fulfilled the inclusion criteria were observational, the Newcastle-Ottawa Scale(NOS) (17) was employed to evaluate the potential for bias in the literature. At the study level, we assessed the risk of bias in articles using the NOS. Studies were classified as high quality (total score 7–9), moderate quality (total score 4–6), or low quality (total score 0–3) (18).

### 2.5 Statistical analysis

Using weighted mean difference (WMD), the difference in serum/plasma HCY levels between the OSAHS group and the control group was determined. Furthermore, the data were further classified into subgroups based on the following criteria: age, ethnicity, BMI, AHI, and research design. Following this, subgroup data were independently evaluated. WMD was also utilized to compare the serum/plasma HCY concentrations of the pre-CPAP and post-CPAP groups. The I^2^ test assessed the heterogeneity of WMDs. When heterogeneity was modest (I^2^< 50), the fixed-effects model was employed; otherwise, the random-effects model was utilized. We will use either the fixed-effect or random-effects models to estimate treatment effects across trials. The fixed-effect model will be used when it is plausible to believe that all studies are estimating the same underlying treatment effect. In addition, for the meta-analysis, we produced a pooled adjusted HR estimate across all studies to investigate the link between HCY concentration and MACCEs risk. To explore the relationships between HCY concentration and AHI scores in patients with OSAHS, Spearman’s correlation coefficients (CORs) were utilized to conduct a meta-analysis. The analysis was executed utilizing the “meta” program in R. It was not expected that Spearman’s product-moment COR would be dependent on the sample distribution, given that the latter often relies on the significance of the rank COR (as indicated by the standard error). Through the implementation of the Fisher transformation, direct comparisons were conducted among all CORs. Following that, the values that had been modified were employed as input values for the analysis. The results were subsequently recalculated as CORs (19). Using Cohen’s criterion, the computed effect size (small, ≤ 0.3; moderate, 0.3–0.5; and large, > 0.5) was evaluated. Furthermore, to examine the correlation between the HCY concentration and AHI scores, Spearman’s COR was utilized. Per the clarification provided, a multitude of scholarly articles have cited the following equation as a technique for transforming Pearson’s COR into Spearman’s COR:


$$r=2\;\text{sin}\;\left(r_{s}\frac{\pi }{6}\right),$$

where *r* and *r_s_*, respectively, represented the CORs computed by Pearson and Spearman’s techniques. To evaluate publication bias, Egger’s tests were executed. The stability of the meta-analysis was assessed d using a sensitivity analysis. STATA software (Release 10.0, Stata Corp LLC, College Station, TX, USA) and R software (Auckland, North Island, New Zealand, R Project for Statistical Computing, http://www.r-project.org/, version 4.1.2) were used for data analysis, and *P*< 0.05 was defined as being statistically significant.

## 3 Results

### 3.1 Results of search

**Figure 1:**
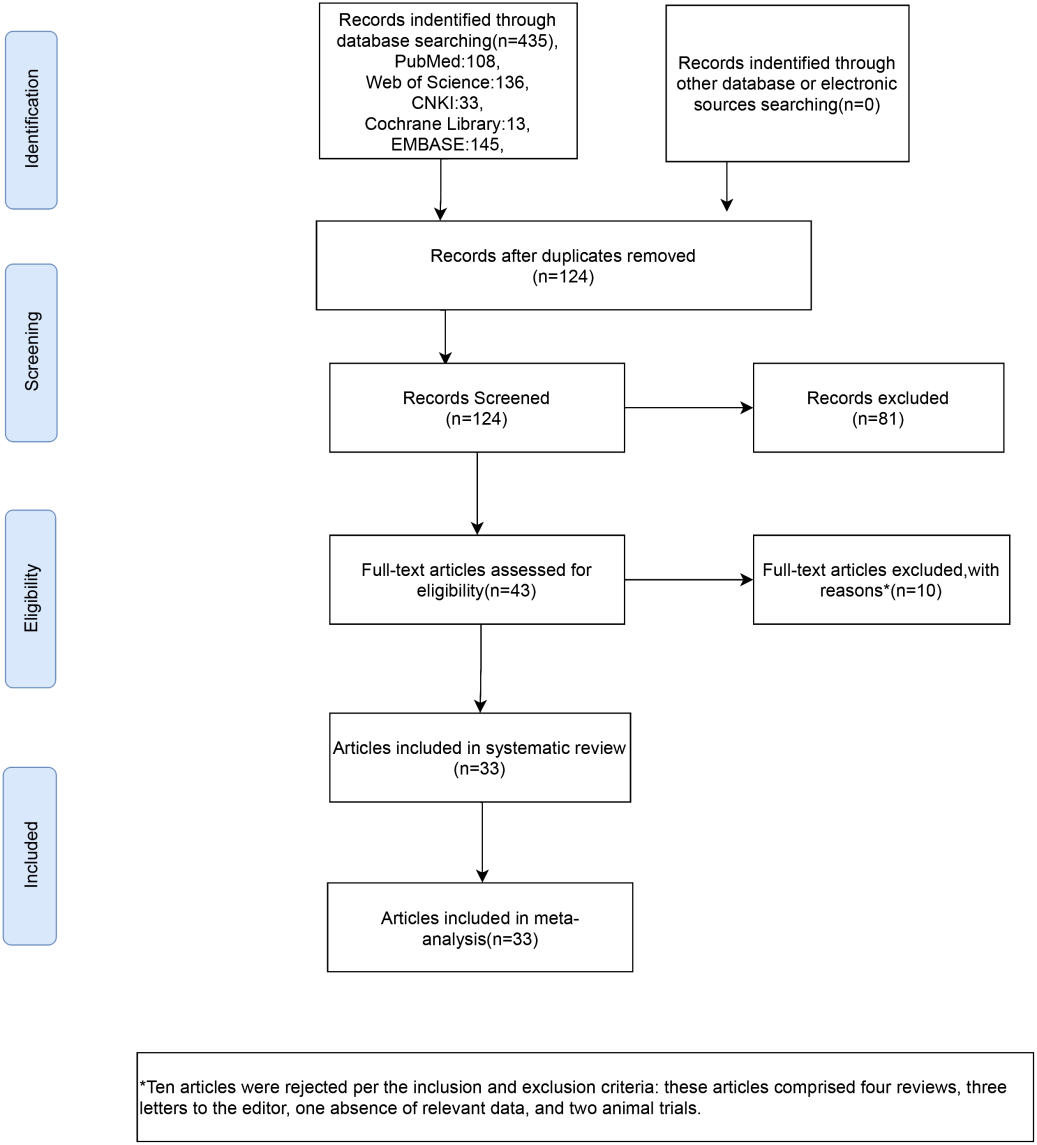
Flow chart of inclusion criteria in the study.

From the database, 435 studies on the subject were extracted in total. 124 articles were identified following the elimination of duplicates. Following a process of abstract and title evaluation to exclude 81 publications deemed seemingly unrelated, the final selection comprised 43 articles. The full texts of these articles were retrieved and thoroughly scrutinized. Ten articles were rejected per the inclusion and exclusion criteria; these articles comprised four reviews, three letters to the editor, one absence of relevant data, and two animal trials. Finally, 33 (7, 11, 12, 20–49)articles were included in our meta-analysis (**Figure 1**).

### 3.2 Characteristics of included studies

Twenty-three articles compared the HCY concentrations of individuals with OSAHS and control individuals. Twelve articles documented HCY concentrations in individuals with OSAHS before and after CPAP therapy. Three publications reported the relationship between HCY concentrations and the occurrence of MACCEs in patients with OSAHS. Ten articles reported the Spearman or Pearson COR between HCY concentration and AHI values. **Table 1** lists the authors, publication year, nation, sample size, sample type, and NOS score for each study. **Table 2** shows the mean age, mean BMI, Pearson/Spearman correlation coefficient, and HCY values for each study.

**Table 1:** Characteristics of included studies.

Author	Year	Country	Sex (M/F)		Age (year)		BMI (kg/m^2^)		HCY source	Study design
			Case	Control	Case	Control	Case	Control		
Lavie L	2001	Israel	127/0	73/0	47.78 ± 11.64	47.82 ± 11.15	30.38 ± 5.50	26.59 ± 3.21	Plasma	Case-control study
Jordan W	2004	Germany	12/4		53.38 ± 12.04		36.87 ± 6.83		Serum	Cross-sectional study
Robinson GV	2004	UK	108	112	49.7 (10.3)	49.1 (10.3)	35.6 (7.6)	35.9 (6.3)	Plasma	Cross-sectional study
Can M	2006	Turkey	62/0	30/0	47.14 ± 1.62	42.6 ± 3.2	29.63 ± 0.67	20.2 ± 0.82	Serum	Case-control study
Kokturk O(a)	2006	Turkey	25/0	42/0	51.76 ± 8.67	51.76 ± 8.67	32.27 ± 4.89	32.27 ± 4.89	Serum	Case-control study
Kokturk O(b)	2006	Turkey	47/0	42/0	48.56 ± 10.02	51.76 ± 8.67	30.53 ± 5.1	32.27 ± 4.89	Serum	Case-control study
Kumor M	2006	Poland	32	12	51.3 ± 10.3	42.8 ± 16.8	30.6 ± 4.4	26.9 ± 2.95	Serum	Case-control study
Ryan S(a)	2007	Ireland	35/0	30/0	42 ± 8	41 ± 6	32.9 ± 6.03	30.7 ± 3.1	Serum	Case-control study
Ryan S(b)	2007	Ireland	31/0	30/0	43 ± 9	41 ± 6	32.1 ± 3.5	30.7 ± 3.1	Serum	Case-control study
Ryan S(c)	2007	Ireland	14/0	30/0	39 ± 9	41 ± 6	42.5 ± 4.8	30.7 ± 3.1	Serum	Case-control study
Steiropoulos P(good compliance)	2007	Greece	16/4		46.8 ± 11.54		36.36 ± 9.8		Serum	Cross-sectional study
Steiropoulos P(poor compliance)	2007	Greece	19/0		44.95 ± 10.08		32.33 ± 5.10		Serum	Cross-sectional study
Ozkan Y	2008	Turkey	28/6	8/7	48.7 ± 11.0	43.5 ± 9.3	30.8 ± 4.7	27.4 ± 4.3	Serum	Case-control study
Wang X	2008	China	81/0	120/0	52.6± 7.7	50.2 ± 10.3	25.5± 2.6	25.1± 3.7	Serum	Case-control study
Yavuz Z	2008	Turkey	62	12	53.3 ± 8.9	5.8 ± 9.3	32.1 ± 3.8	30.8 ± 4.0	Serum	Case-control study
Sariman N	2010	Turkey	23/15		49 ± 12		31.27 ± 5.24		Plasma	Cross-sectional study
Wang L(old)	2010	China	30/2	27/2	65.8 ± 7.2	69.4 ± 4.2	23.34 ± 2.36	26.85 ± 2.9	Serum	Case-control study
Wang L(young)	2010	China	46/5	20/3	42.7 ± 8.3	44.7 ± 12.3	28.36 ± 3.51	25.13 ± 3.61	Serum	Case-control study
Basoglu OK	2011	Turkey	23/13	17/17	50.0 ± 19.7	49.7 ± 11.1	33.5 ± 5.7	34.5 ± 2.9	Serum	Case-control study
Chen M(a)	2011	China	11/9	15/12	58.65 ± 10.36	58.78 ± 10.34	24.81 ± 1.79	22.01 ± 2.30	Serum	Cross-sectional study
Chen M(b)	2011	China	19/12	15/12	63.74 ± 9.72	58.78 ± 10.34	25.85 ± 2.27	22.01 ± 2.30	Serum	Cross-sectional study
Chen M(c)	2011	China	15/9	15/12	66.79 ± 8.97	58.78 ± 10.34	26.40 ± 2.96	22.01 ± 2.30	Serum	Cross-sectional study
Cintra F	2011	Brazil	35/40	35/40	53.18 ± 6.72	53.25 ± 6.55	31.02 ± 6.67	27.24 ± 4.17	Serum	Case-control study
Cintra F(1 month group)	2011	Brazil	14	15			26.4 ± 7.2	28.0 ± 9.1	Serum	Case-control study
Cintra F(6 month group)	2011	Brazil	14	15			25.5 ± 8.8	28.0 ± 9.1	Serum	Case-control study
Kumor M (OSAHS with ischemic heart disease)	2011	Poland	23						Plasma	Cross-sectional study
Kumor M (pure OSAHS)	2011	Poland	42						Plasma	Cross-sectional study
Monneret D	2012	France	17/9	3/6	61.5 ± 5.0	59.7 ± 3.4	29.8 ± 3.7R	29.1 ± 2.6	Serum	Cross-sectional study
Sales LV	2013	Brazil.	14/0	13/0	37.2 ± 6.9	36.0 ± 6.1	28.8 ± 3.3	26.9 ± 2.9	Serum	Cross-sectional study
Dal-Fabbro C	2014	Brazil	24/5		47.0 ± 8.9		28.4 ± 3.6		Plasma	
Andaku DK	2015	Brazil	11/0	10/0	42.36 ± 9.48	43.00 ± 10.56	24.14 ± 2.67	26.65 ± 2.38	Serum	Cross-sectional study
Monneret D	2016	France	28/0		48.9 ± 9.4		26.7 ± 3.1	26.6 ± 2.8		
Feliciano A	2017	Portugal	73/0	30/0	46 ± 8	45 ± 10	30.2 ± 3.7	27.4 ± 3.3	Serum	Case-control study
Li J(a)	2018	China	37/4	29/4	42.6 ± 8.9	44.7 ± 10.1	24.36 ± 3.61	25.08 ± 3.35	Serum	Case-control study
Li J(b)	2018	China	33/3	29/4	40.5 ± 12.3	44.7 ± 10.1	26.49 ± 2.78	25.08 ± 3.35	Serum	Case-control study
Li J(c)	2018	China	35/5	29/4	41.9 ± 8.9	44.7 ± 10.1	27.34 ± 2.82	25.08 ± 3.35	Serum	Case-control study
Zhu X(a)	2018	China	22	50	65.12 ± 6.35				Serum	Case-control study
Zhu X(b)	2018	Chian	23	50	65.12 ± 6.35				Serum	Case-control study
Zhu X(c)	2018	China	41	50	65.12 ± 6.35				Serum	Case-control study
Ko CY(a)	2019	China	35/5	11/9	45.4 ± 11.0	39.0 ± 8.9	26.98 ± 4.72	24.31 ± 2.25	Serum	Case-control study
Ko CY(b)	2019	China	18/4	11/9	48.6 ± 13.8	39.0 ± 8.9	26.04 ± 3.69	24.31 ± 2.25	Serum	Case-control study
Ko CY(c)	2019	China	27/3	11/9	44.4 ± 11.2	39.0 ± 8.9	29.04 ± 4.79	24.31 ± 2.25	Serum	Case-control study
Plociniczak A	2019	Poland	13/44	21/23	58 (48–63)	52 (42–58)	31.1 (28.6–35.0)	26.3 (23.9–29.7)	Serum	Case-control study
Li N	2021	China	1611/739		49.45 ± 10.65				Plasma	Cohort study
Liu L	2022	China	661/439		66(60-96)				Plasma	Cohort study
Tang SS	2022	China	17/9	21/6	45.58 ± 8.81	47.59 ± 5.18	24.63 ± 2.61	27.03 ± 2.11	Serum	Case-control study
Chen X	2023	China	716/837	595/139	56.3 ± 10.5		34.2 ± 5.8		Serum	Cohort study
Chiu LW	2023	Taiwan	247/14	247/14			26.6 ± 3.6	27.7 ± 3.9	Serum	Cross-sectional study
Gungordu N	2023	Turkey	24/6		46.5 ± 9.8		34.2 ± 5.8		Serum	Cross-sectional study
Polonsky EL	2023	Russia	84	68	55.3 ± 12.0	49.2 ± 10.9	32.48 ± 5.72	27.32 ± 4.22	Serum	Case-control study
Xia W	2023	China	38/7	22/39	70.62 ± 5.90	68.27 ± 5.51	27.32 ± 3.85	21.27 ± 5.90	Serum	Case-control study

a: Mild; b: Moderate; c: Severe; ELISA, enzyme linked immunosorbent assay; F, female; M, male; BMI, body mass index; HCY, Homocysteine.

**Table 2:** Participants’ characteristics of included studies.

Author	Year	Mean (SD) HCY, µmol/l		Mean (SD) HCY, µmol/l		Spearman cor	HR 95%CI	NOS
		Case	Control	Pre-CPAP	Post-CPAP			
Lavie L	2001	9.85 ± 2.99	9.78 ± 3.49					8
Jordan W	2004	8.87 ± 2.26	7.64 ± 3.06	9.64 ± 3.83	8.01 ± 3.13			7
Robinson GV	2004	9.90 ± 3.20	9.26 ± 3.80					8
Can M	2006	21.53 ± 14.20	6.80 ± 4.70			R=0.30		8
Kokturk O(a)	2006	17.2 ± 6.58	10.35 ± 3.63			R=0.73		6
Kokturk O(b)	2006	14.7 ± 4.52	10.35 ± 3.63			R=0.53		6
Kumor M	2006	8.20 ± 2.90	9.30 ± 2.10					8
Ryan S(a)	2007	7.16 ± 1.58	8.30 ± 2.12			R=0.21		8
Ryan S(b)	2007	8.40 ± 2.98	8.30 ± 2.12			R=0.21		8
Ryan S(c)	2007	9.16 ± 4.87	8.30 ± 2.12			R=0.21		8
Steiropoulos P(good compliance)	2007			12.30 ± 1.90	10.98 ± 1.51			8
Steiropoulos P(poor compliance)	2007			14.72 ± 5.83	12.77 ± 3.58			8
Ozkan Y	2008	16.40 ± 5.70	11.20 ± 1.90			R=0.80		6
Wang X	2008	14.00 ± 2.90	11.50± 2.30					6
Yavuz Z	2008	13.50 ± 6.00	10.20 ± 2.90					8
Sariman N	2010					R=0.18		7
Wang L(old)	2010	18.70 ± 4.73	11.13 ± 3.05			R=0.30		8
Wang L(young)	2010	10.84 ± 2.53	8.90 ± 1.23			R=0.30		8
Basoglu OK	2011	18.10 ± 2.70	17.90 ± 4.50					8
Chen M(a)	2011	11.46 ± 3.31	8.98 ± 3.74			R=0.48		7
Chen M(b)	2011	14.18 ± 4.36	8.98 ± 3.74			R=0.48		7
Chen M(c)	2011	18.57 ± 4.56	8.98 ± 3.74			R=0.48		7
Cintra F	2011	15.11 ± 3.76	15.43 ± 3.58					8
Cintra F(1 month group)	2011			14.00 ± 3.10	14.80 ± 4.40			8
Cintra F(6 month group)	2011			14.00 ± 3.10	13.90 ± 3.60			8
Kumor M(pure OSAHS)	2011			11.65 ± 2.40	11.30 ± 3.70			6
Kumor M(OSAHS with ischaemic heart disease)	2011			12.40 ± 3.80	10.90 ± 3.20			6
Monneret D	2012	12.80 ± 3.80	9.50 ± 2.50			R=0.52		7
Sales LV	2013	16.70 ± 8.00	10.7 ± 2.9					8
Dal-Fabbro C	2014	10.30 ± 1.50	9.30 ± 1.10					7
Andaku DK	2015	10.75 ± 1.07	15.09 ± 1.31					8
Monneret D	2016	11.90 ± 0.80	11.2 ± 0.60					7
Feliciano A	2017	15.30 ± 3.50	14.70 ± 3.30					6
Li J(a)	2014	10.30 ± 2.00	8.70 ± 0.70			R=0.13		7
Li J(b)	2014	13.30 ± 1.60	8.70 ± 0.70			R=0.13		7
Li J(c)	2014	9.50 ± 1.80	8.70 ± 0.70			R=0.13		7
Zhu X(a)	2018	18.62 ± 3.29	11.66 ± 0.71					6
Zhu X(b)	2018	26.34 ± 4.05	11.66 ± 0.71					6
Zhu X(c)	2018	32.28 ± 3.92	11.66 ± 0.71					6
Ko CY(a)	2019	15.99 ± 6.33	12.50 ± 4.31					7
Ko CY(b)	2019	17.32 ± 10.57	12.50 ± 4.31					7
Ko CY(c)	2019	16.41 ± 6.50	12.50 ± 4.31					7
Plociniczak A	2019	14.6 (11.9–17.2)	15.8 (10.8–18.2)					8
Li N	2021						1.35(1.08,1.65)	8
Liu L	2022						4.78(2.10,10.93)	7
Tang SS	2022	15.11 ± 7.68	10.18 ± 3.32					7
Chen X	2023						1.36(1.02,1.83)	7
Chiu LW	2023			12.1 ± 3.9	11.4 ± 3.7	R=0.26;		6
Gungordu N	2023	13.70 ± 3.70	13.80 ± 4.60					6
Polonsky EL	2023	14.28 ± 5.13	13.07 ± 5.15					7
Xia W	2023	29.71 ± 6.27	12.50 ± 4.19					8

a: Mild; b: Moderate; c: Severe; R, correlation coefficient between HCY levels and AHI; cor, correlation coefficients.

### 3.3 Meta-analysis

#### 3.3.1The difference in HCY concentration between controls and patients with OSAHS

**Figure 2:**
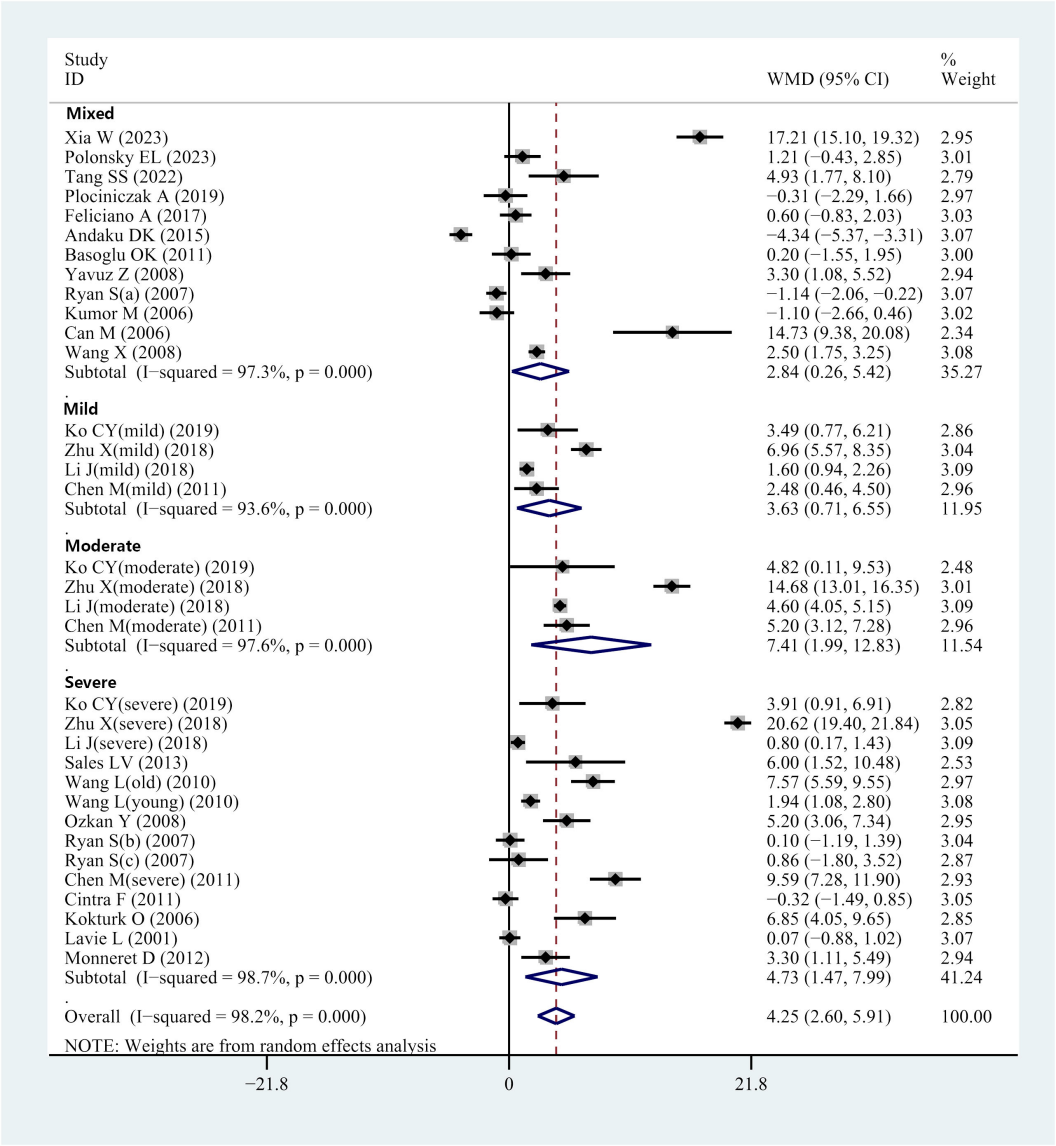
Forest plot of WMD and its 95%CI for HCY levels in integral OSAHS patients group compared to the control group in meta-analysis.

The I^2^ value was 98.2%, indicating high heterogeneity of the study. Therefore, the effect values were combined using a random effects model. The results of the meta-analysis indicated that the OSAHS group had serum/plasma HCY levels 4.25 μmol/L higher than the control group (WMD = 4.25 μmol/L, 95% CI: 2.60–5.91, *P<* 0.001) (**Figure 2**).

#### 3.3.2 Subgroup analysis-disease severity

Several of the included studies revealed serum or plasma HCY levels in patients with OSAHS of various severity. This prompted subgroup analyses according to mild, moderate, or severe OSAHS. Four studies provided data on serum/plasma HCY levels in individuals with moderate OSAHS. Results indicated elevated HCY levels in individuals with mild OSAHS compared with controls (WMD = 3.63 μmol/L, 95% CI: 0.71–6.55, *P* = 0.015) (**Figure 2**). Furthermore, data regarding serum/plasma HCY levels were accessed from 4 studies that comparatively assessed individuals with moderate OSAHS and controls. HCY levels in the serum and plasma were higher in patients with moderate OSAHS than in the control group (WMD = 7.41 μmol/L; 95 percent CI: 1.99–2.83; *P* = 0.007) (**Figure 2**). Additionally, 14 studies reported data regarding serum/plasma HCY levels of individuals with severe OSAHS and controls. The results revealed an elevation in plasma/serum HCY levels in individuals with severe OSAHS compared with controls (WMD = 4.73 μmol/L, 95% CI: 2.60–5.91, *P* = 0.004) (**Figure 2**). Twelve studies did not strictly differentiate the participant population in terms of severity, which was defined as a mixed group. Nevertheless, the OSAHS group had increased HCY levels compared to the control group (WMD = 2.84 μmol/L, 95% CI: 0.26–5.42, *P* = 0.031) (**Figure 2** and **Table 3**).

**Table 3:** Subgroup analyses of the association between homocysteine levels and OSAHS in this meta-analysis.

Subgroup	No. of studies	WMD	95%CI	P for heterogeneity	I^2^(%)	P value between groups
Ethnicityz						
Asian	17	6.65	4.16,9.15	<0.001	98.7	<0.001
Caucasian	17	1.46	0.16,2.77	<0.001	91.0	0.028
Disease severity						
Mild	4	3.63	0.71, 6.55	<0.001	93.6	0.015
Moderate	4	7.41	1.99,12.83	<0.001	97.6	0.007
Severe	14	4.73	1.47,7.99	<0.001	98.7	0.004
Mean BMI						
≥30	13	0.94	0.05,1.92	<0.001	81.2	0.041
<30	21	0.28	0.1,0.46	<0.001	98.7	<0.001
Research style						
Case-control study	28	4.39	2.59,6.19	<0.001	98.3	<0.001
Cross-sectional study	6	3.64	-1.25, 8.50	0.145	97.2	<0.001
Mean age						
≥65	9	9.51	4.62,14.4	<0.001	98.6	<0.001
<65	25	2.00	0.97, 3.03	<0.001	94.0	<0.001

WMD, weighted mean difference.

#### 3.3.3 Subgroup analysis-mean BMI

Most of the included studies provided BMI data. We performed subgroup analyses based on whether the mean BMI was ≥ 30 kg/m^2^ to determine whether BMI affected HCY levels. Results of analysis revealed elevated serum/plasma HCY levels among individuals with OSAHS compared with controls in both subgroups (i.e., BMI > and< 30 kg/m^2^); those with a mean BMI ≥ 30 kg/m^2^, WMD = 0.94 μmol/L (95% CI: 0.05–1.92, *P =* 0.041), whereas those with a mean BMI< 30 kg/m^2^ exhibited an WMD = 6.24 μmol/L (95% CI: 3.88–8.60, *P<* 0.001) (**Table 3**).

#### 3.3.4 Subgroup analysis-mean age

We performed subgroup analysis based on whether the mean age was ≥ 65 years. Results indicated that HCY concentrations were greater in the OSAHS subgroup with a mean age of< 65 years compared to the control group (WMD = 2.00 μmol/L, 95% CI: 0.97–3.03, *P*< 0.001) and in the subgroup with mean age ≥ 65 years (WMD = 9.51 μmol/L, 95% CI: 4.62–14.40, *P*< 0.001) (**Table 3**).

#### 3.3.5 Subgroup analysis-ethnicity

Subgroup analysis was performed to explore the possible sources of heterogeneity. Participants were divided into groups based on their ethnicity: Asian (n = 17 studies) and Caucasian (n = 17 studies). Compared to the control group, the OSHAS group’s serum/plasma HCY levels were higher among Asians (WMD = 6.65 μmol/L; 95% CI]: 4.16–9.15; *P*< 0.001). In the Caucasian population, these HCY levels exhibited the same pattern (WMD = 1.46 μmol/L, 95% CI: 0.16–2.77, *P* = 0.028) (**Table 3**).

#### 3.3.6 Subgroup analysis-study design

Subgroup analyses were conducted per the study design to control for any heterogeneity that might result from variations in study designs. In the study, 28 case-control studies were considered. Overall results showed that participants with OSAHS had elevated serum/plasma HCY levels in comparison to the control group (WMD = 4.39 μmol/L, 95% CI: 2.59–6.19, *P*< 0.001). Furthermore, the results of the six cross-sectional investigations showed that there was no discernible difference between individuals with OSAHS and controls’ serum/plasma HCY levels (WMD = 3.64 μmol/L; 95% CI: -1.25–8.50; *P* = 0.145) (**Table 3**).

### 3.4 Sensitivity analysis

**Supplementary Figure 1A** displays the plots corresponding to the “one-study-removed” analysis. These analyses offer significant insights into the consistency of the pooled data concerning plasma/serum HCY levels in patients with OSAHS compared to the control group.

### 3.5 Publication bias

Funnel plots were used to analyze publication bias in studies that investigated variations in serum/plasma HCY levels between controls and patients with OSAHS. The funnel plot presented in **Supplementary Figure 1B** exhibited symmetry, indicating the absence of publication bias, which was supported by statistical analysis, as evidenced by the results of Egger’s test. The calculated *t*-value of 1.35 and *P*-value of 0.188 indicated no statistically significant publication bias.

### 3.6 Comparison of serum/plasma HCY concentrations before and after CPAP treatment in patients with OSAHS

**Figure 3:**
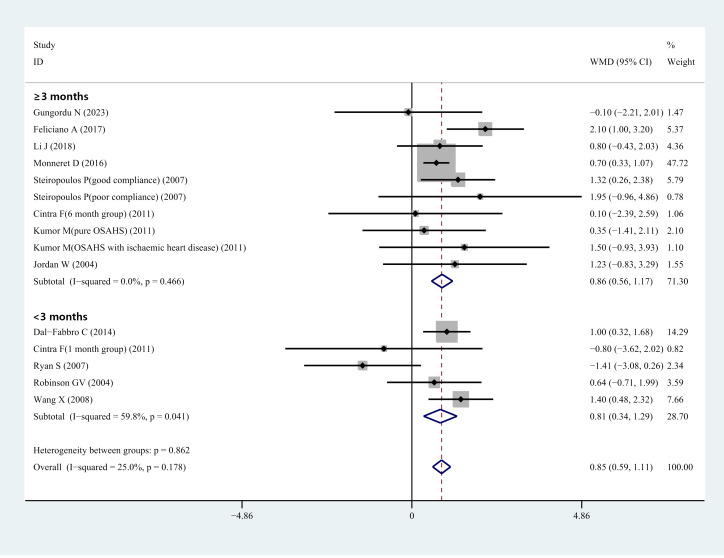
Forest plot of WMD and its 95%CI for HCY levels in pre-CPAP treatment group compared to post-CPAP treatment group in meta-analysis.

Fifteen studies included data from 464 patients with OSHAS. One study (50) compared HCY levels before and after therapy for individuals with OSAHS and OSAHS paired with ischemic heart disease. One article (51) presented data on HCY concentrations in the 1-month and 6-month treatment groups, respectively. One article (42) reported treatment outcome data for the good adherence group and the poor adherence group, respectively. Our analysis yielded I^2^ = 25%, P = 0.178, showing that there was no heterogeneity in these investigations. We utilized a fixed-effects model to integrate effect sizes. The meta-analysis found that the overall WMD for HCY levels was 0.85 μmol/L (95% CI: 0.59–1.11, *P*< 0.001) (**Figure 3**). In light of this, we concluded that CPAP treatment might substantially lower serum/plasma HCY levels when all relevant studies were included. We performed subgroup analyses based on treatment duration. Duration of treatment< 3 months: the combined WMD value for studies with a mean follow-up of< 3 months was not statistically significant, corresponding to a value of 0.52 μmol/L (95% CI: -0.36–1.39, *P<* 0.001) (**Figure 3**). Duration of treatment ≥ 3 months: total WMD was statistically significant in studies with a mean follow-up of more than 3 months, corresponding to a value of 0.85 μmol/L (95% CI: 0.56–1.17, *P<* 0.001) (**Figure 3**). Thus, the analysis showed no significant decrease in HCY levels while the treatment lasted< 3 months, but a significant decrease could be observed after 3 months of treatment. We performed subgroup analyses based on the mean BMI. In studies with a mean BMI< 30 kg/m^2^, the total WMD value was statistically significant, corresponding to a value of 0.81 μmol/L (95% CI: 0.51–1.10, *P<* 0.001). In studies with mean BMI ≥ 30 kg/m^2^, total WMD values were statistically significant, corresponding to a value of 0.98 μmol/L (95% CI: 0.46-1.50, *P*<0.001). The analysis showed that serum/plasma HCY levels decreased after CPAP treatment in patients with OSAHS regardless of whether the BMI was ≥ 30 kg/m^2^ or< 30 kg/m^2^ (**Supplementary Figure 2**).

### 3.7 Sensitivity analysis

Sensitivity analysis of each outcome index revealed that there was minimal difference between the meta-results analyses before and after the removal of each study individually, as well as minimal difference between the meta-results analyses after model transformation (**Supplementary Figure 3A**).

### 3.8 Publication bias

Neither funnel plots (**Supplementary Figure 3B**) nor Egger tests indicated publication bias (*t* = -0.25; *P* = 0.807).

### 3.9 The relationship between high HCY concentrations and the occurrence of MACCEs in patients with OSAHS

**Figure 4:**
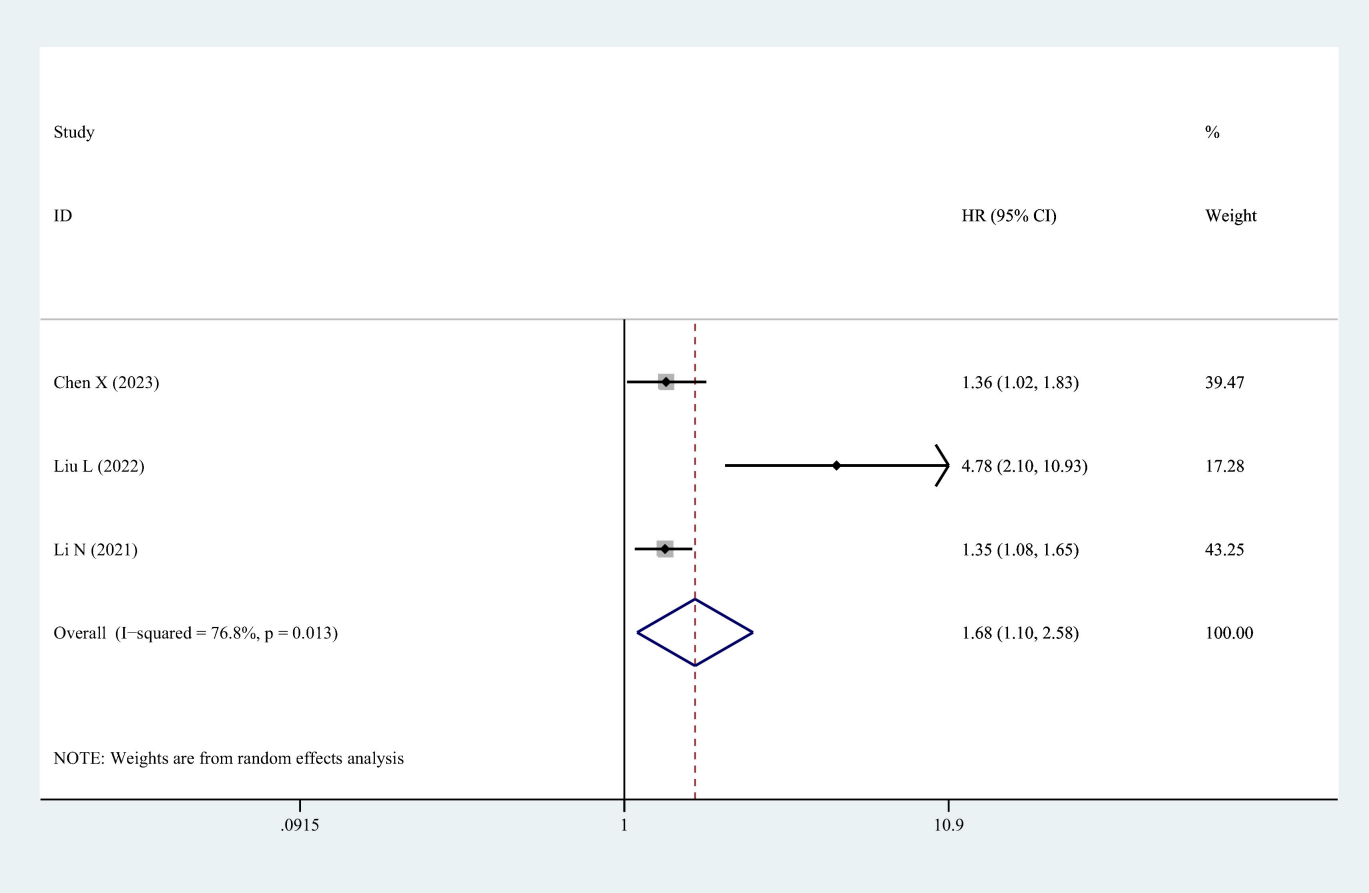
The forest plot of the relationship between HCY levels and major adverse cerebrocardiovascular events risk in patients with OSAHS.

Three studies identified high serum/plasma HCY concentration levels as an independent risk factor for the occurrence of MACCEs in patients with OSAHS, and they reported adjusted HR values. The results of the meta-analysis are expressed as adjusted HR and the corresponding 95% CI. The findings of combining the adjusted HR values from these studies suggested that serum/plasma HCY levels and MACCEs were significantly correlated. The overall HR was calculated to be 1.68 (95% CI 1.10–2.58; *P* = 0.017), as illustrated in **Figure 4**.

### 3.10 Relationship between AHI scores and serum/plasma HCY levels

**Figure 5:**
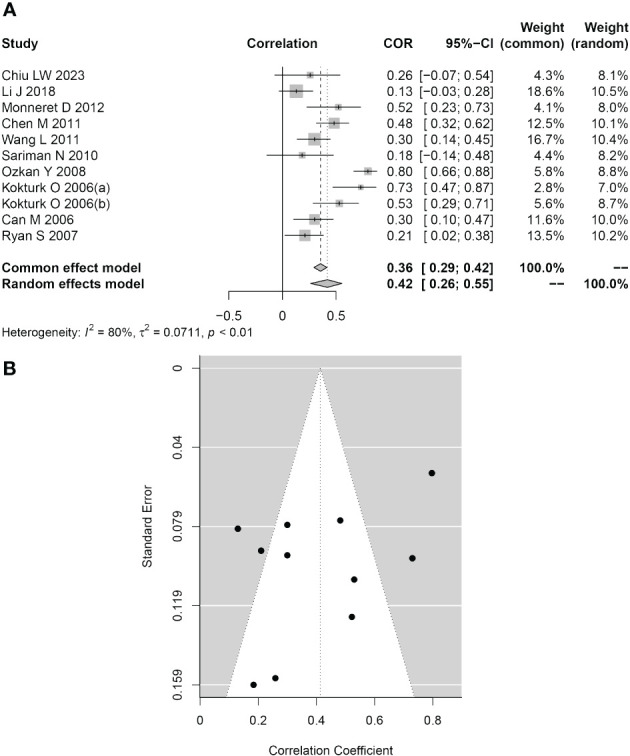
Association between HCY levels and the AHI. **(A)** Forest plot for the relationship between HCY levels and the AHI. **(B)** Funnel plot of effect sizes measured as correlations between HCY levels and the AHI.

Eleven studies reported Pearson or Spearman COR values between plasma/serum HCY levels and AHI scores. The AHI score is a crucial metric for assessing the severity of OSAHS. We examined the connection between serum/plasma HCY levels and AHI scores in the included group using the “meta” R package since we believed that these levels would be related to the severity of OSAHS. As seen in **Figure 5A**, the analysis’s findings revealed a moderately significant correlation (COR = 0.42, 95% CI 0.26–0.55; *P*< 0.001) between the two variables. We also assessed the aforementioned studies’ publication biases. **Figure 5B** depicts a symmetrical funnel plot, and the Egger test *P* value (*P* = 0.065) was not statistically significant.

## 4 Discussion

The connection between serum/plasma HCY and OSAHS has received much attention; however, there is controversy about comparing serum/plasma HCY levels between individuals with OSAHS and the general population. Patients with OSAHS had higher serum/plasma HCY levels than controls, particularly in instances of moderate to severe OSAHS, according to our meta-analysis. In patients with OSAHS treated with 3 months of CPAP, serum/plasma HCY levels were significantly reduced. Furthermore, HCY levels may be strongly integrated with the risk of MACEs in patients with OSAHS. HCY levels were also favorably correlated with the AHI ratings. In the sensitivity analyses, the combined results were not affected after deleting a study item by item. Therefore, the results of our meta-analysis were highly reliable.

Important inflammatory variables in cardiovascular disease include HCY. During the conversion of the important amino acid methionine to cysteine, this sulfur-containing amino acid is produced (52). HCY is a non-essential alpha-amino acid that does not contribute to the production of proteins. Various forms of HCY are found in the systemic circulation: approximately 70%–80% is bound to plasma proteins; 20%–30% is coupled to other HCY molecules to form disulfides; and a negligible fraction is found as free thiols (1%–2%) (53). In general, decreased concentrations of HCY indicate a physiological state that is clinically stable and devoid of any active inflammation (54). Deficiencies in folic acid, vitamin B6, and vitamin B12 may result in increased levels of HCY (55). Folic acid and these B vitamins are needed for HCY metabolism. Due to the similarities shared by OSAHS and HCY in terms of oxidative stress and chronic inflammatory states, recent studies have also discussed the relationship between OSAHS and elevated HCY levels. Some studies have shown that recurrent apnea during sleep in patients with OSAHS leads to hypoxemia, which promotes the release of various active substances, which in turn stimulates the release of endothelin-1 from endothelial cells, causes oxidative stress, reduces nitric oxide activities, and affects the remethylation process of HCY metabolism, resulting in abnormally elevated serum HCY levels (56). In the meantime, Monneret et al. (34) showed that serum HCY levels were proportionate to the severity of OSAHS and higher in patients with OSAHS with metabolic syndrome than in patients with OSAHS without metabolic syndrome. Our latter results revealed that CPAP therapy significantly lowered serum/plasma HCY levels in patients with OSAHS. Cintra et al. (51) found no change in plasma HCY levels between age- and sex-matched patients with OSAHS and controls. According to Chen et al. (22), advanced age, obesity, renal insufficiency, thyroid illness, genetic background, environmental variables, and other drugs all have a greater influence on HCY levels than OSAHS itself. Therefore, we conducted subgroup analyses depending on age, mean, BMI, ethnicity, and study design, and the results were consistent: patients with OSAHS had higher serum/plasma levels than controls. The inconsistency between the results of individual studies and meta-analyses may be due to the small sample size of individual studies and the inconsistent criteria of the included population, which may lead to biased results. However, due to the low quantity and quality of included studies, the results drawn from these studies’ analyses must be supported by larger samples and high-quality investigations.

To enhance our comprehension of the time required for CPAP treatment to decrease HCY levels, we conducted a subgroup analysis predicated on treatment duration. HCY levels decreased significantly 3 months after therapy, but a non-significant decline was observed within 3 months. This is reinforced by the findings of Chen et al.’s (57) meta-analysis. Consistent with our findings, Jordan et al (27) discovered that continuous CPAP usage can successfully lower HCY levels by approximately 30% in patients with OSAHS and minimize the risk of cardiovascular disease. Nevertheless, a study (38) found that the reduction in HCY levels was not statistically significant after 6 months of CPAP treatment. This may be attributed to the complexity of the variables that influence the efficacy of CPAP on HCY levels, such as patient adherence, variations in their physical condition, and the type and parameters of the noninvasive ventilator. In our investigation, we were unable to ascertain the precise duration of time that CPAP treatment decreased HCY levels in individuals with OSAHS. It may be related to HCY metabolism and patient compliance. We believe, however, that HCY levels can be decreased with long-term CPAP therapy (> 3 months), given the evidence currently available. One study (58) reported that women with overweight or obesity showed significantly higher HCY levels (respectively, 6.6 and 6.3 µmol/L) compared to women with normal BMI (Hcy levels: 6.1 µmol/L). Some data showed a negative correlation between change in total homocysteine levels and weight loss, suggesting that people who lost more weight (> 10 kg) showed an increase in total homocysteine (59). Furthermore, to determine whether BMI influences the efficacy of CPAP treatment, we performed a subgroup analysis based on BMI. The results indicated that CPAP treatment decreased HCY levels in patients with OSAHS, irrespective of BMI ≥ 30 kg/m^2^. This may be because nocturnal hypoxemia in patients with OSAHS is alleviated by CPAP, which maintains an open airway by delivering positive pressure to the airway of spontaneously breathing individuals throughout the respiratory cycle. Transoral robotic surgery decreases HCY levels in patients with OSAHS, particularly in patients with severe OSAHS, as demonstrated by Chiu LW. et al. (23). They believed that OSAHS would continue to improve if the clogged upper airway of individuals with OSAHS was successfully cleared. Our results indicate that serum/plasma HCY concentrations are positively correlated with the AHI index; this conclusion is consistent with those of earlier researchers.

Recent research has established HCY as a distinct risk factor for cardiovascular disease (60). From a pathophysiological perspective, it induces cardiovascular events by causing dysfunction in the endothelium and irregularities in blood coagulation. Elevated plasma HCY levels are linearly correlated with cardiac autonomic dysfunction in patients with OSAHS (61). Bouchey et al. (62) found that for every 5 mmol/L elevation in plasma HCY, there was an increased risk of coronary artery disease by 60%–80% and a 50% increase in the incidence of cerebrovascular disease. John et al. (27) found that elevated HCY may account for 10% of cardiovascular disease risk and that reducing HCY levels reduced cardiovascular risk by 25%. This meta-analysis matches the aforementioned research. Elevated HCY levels may be linked to oxidative stress, endothelial dysfunction, and vascular remodeling. HCY induces the expression of E-selectin, monocyte chemotactic protein-1, and vascular cell adhesion molecule-1. This leads to the adhesion of monocytes to the endothelium of arteries and may significantly promote the development of atherosclerosis by facilitating macrophage infiltration of the arterial wall (63–65). It has been shown that HCY can contribute to the increased uptake of low-density lipoprotein (LDL) by macrophages, as well as oxidize LDL and increase foam cells, which are involved in the atherosclerotic process (66). Elevated levels of HCY are involved in the onset and progression of vascular disease. During a 6-year follow-up period, a study found that the prevalence of hypertension was significantly higher in patients with OSAHS who had higher HCY levels. This finding may be related to the increased expression of HCY in OSAHS patients. Additionally, the oxidized sulfhydryl group of HCY can produce powerful reactive oxygen species and can also produce superoxide and hydrogen peroxide in circulating metabolism. Oxidative stress can produce direct vascular cell and tissue biological damage, resulting in thickening of the vascular intima, lumen narrowing, and sclerosis, and promoting the formation and development of hypertension (67). Meanwhile, oxidative free radicals lead to decreased production and bioavailability of endothelium-derived nitric oxide and increased endothelin-1, which leads to impaired endothelium-dependent vascular reactivity and increases the risk of cardiovascular and cerebrovascular accidents (68). Hyperhomocysteinemia has been demonstrated in the past to significantly raise the risk of stroke, particularly in those with hypertension (69). Li et al. (32) showed that in hypertensive patients with OSAHS, plasma HCY concentration was independently correlated with the risk of the first stroke. These results imply that serum/plasma HCY levels might be useful biomarkers for tracking the onset and course of cardiovascular illness in patients with OSAHS. Is it feasible to lower HCY in patients with OASHS and treat them with vitamin B12 and folic acid supplements to lessen their risk of cardiovascular disease? This new idea of preventive treatment deserves more in-depth study.

In meta-analyses, heterogeneity between studies is typically associated with variables including study design, demographic characteristics, and the caliber of included studies. Subgroup analyses based on disease severity, mean age, mean BMI, ethnicity, and type of research design were performed to investigate potential causes of heterogeneity. Unfortunately, no significantly lower I^2^ values were found by subgroup analysis, suggesting that none of these factors mentioned above was a source of heterogeneity. In addition, sensitivity analyses using single-study removal methods did not reveal that a particular study caused high heterogeneity. We hypothesize that other factors may contribute to the source of heterogeneity, such as experimental conditions, HCY testing methods, time of blood sample collection, method of sample storage, gender, smoking status, and lifestyle, all of which can be confounding factors affecting HCY levels.

### 4.1 Strengths and limitations

In a recent meta-analysis, Li et al. (8) and Chen et al. (57) showed that patients with OSAHS had higher HCY levels than the control group and that CPAP therapy could substantially lower HCY levels in patients with OSAHS. Their outcomes agreed with the findings of this investigation. However, this year has seen the emergence of more research on the connection between HCY and OSAHS, and the stronger evidence comes from a larger sample size. Furthermore, the association between HCY and OSAHS severity has not yet been directly shown, nor was the link between HCY levels and OSAHS consequences examined in earlier research. There are several innovations in this study compared to previous meta-analyses: first, this is the most extensive meta-analysis of relevant literature, and subgroup analyses were undertaken to produce more credible results. We considered recently published studies including Chinese people in our meta-analysis. Second, we analyzed the relationship between high HCY concentrations and the occurrence of MACCEs in patients with OSAHS, which helps clinicians assess the risk of serious complications in OSAHS. Again, by quantifying the relationship between HCY levels and AHI scores, primary care physicians can initially assess the severity of a patient’s disease based on HCY and make clinical decisions accordingly. The inclusion of all articles of moderate to high quality enhanced the feasibility of our study. The absence of substantial publication bias implies that the pooled findings can be dependable.

However, it is also important to recognize the specific limitations of this study. First, OSAHS occurs in all ages of children, most commonly in preschool children,with a prevalence of approximately 2% (70). However, the included articles did not focus on the serum/plasma HCY concentrations in children with OSAHS. Thus, the study population included adults with OSAHS, and therefore more attention needs to be paid to the correlation between HCY levels and OSAHS in specific populations (e.g., children with OSAH). Second, this study was primarily a case-control and cross-sectional study that could not reliably establish a causal relationship between OSAHS and HCY. Furthermore, previously published results indicate that gender influences serum HCY, that estrogen lowers blood concentrations of vitamin B12 and folate, and that HCY levels in women rise considerably at 50 years of age (71–74). However, since the inclusion of literature has not been reported for serum/plasma HCY levels in female patients with OSAHS, it is difficult for us to determine whether HCY in patients with OSAHS is affected by gender. As a result, further research is required to explain the findings in this domain.

## 5 Conclusion

In conclusion, serum/plasma HCY levels are significantly increased in patients with OSAHS, and there is a moderately positive correlation between HCY and the AHI scores. HCY levels may be decreased by long-term CPAP treatment in patients with OSAHS. Patients with OSAHS may also have an increased risk of MACCEs due to elevated HCY. Clinical indicators of HCY levels may be utilized to evaluate the severity of OSAHS and the effectiveness of therapy. Considering the heterogeneity of the studies included in this study, future studies should use gender-specific HCY reference values and harmonized HCY testing methods when establishing the relationship between HCY and OSAHS. Further studies with large and homogeneous gender samples could help validate our results.

## Data availability statement

The original contributions presented in the study are included in the article/**Supplementary Material**. Further inquiries can be directed to the corresponding author.

## Author contributions

JH: Conceptualization, Data curation, Formal analysis, Funding acquisition, Investigation, Methodology, Project administration, Resources, Software, Supervision, Validation, Visualization, Writing – original draft, Writing – review & editing. HZ: Data curation, Software, Writing – original draft. JX: Formal analysis, Methodology, Software, Supervision, Validation, Writing – review & editing. YH: Formal analysis, Software, Writing – original draft. NH: Software, Writing – review & editing. JJ: Methodology, Validation, Writing – review & editing.

## Supplementary material

Supplementary FIGURE 1 Sensitivity analysis and funnel plots for the difference in HCY levels between controls and patients with OSAHS. **(A)** Sensitivity analysis, **(B)** Funnel plots of Begg’s test.

Supplementary FIGURE 2 Subgroup analysis depended on mean BMI of pre-CPAP treatment group compared to post-CPAP treatment group in meta-analysis. The means of expression are WMD and its 95%CI.

Supplementary FIGURE 3
**Supplementary Figure 1** Sensitivity analysis and funnel plots for the difference in HCY levels between pre-CPAP treatment group and post-CPAP treatment group. **(A)** Sensitivity analysis, **(B)** Funnel plots of Begg’s test.
