# Supplementary figures and images for: Association between elevated homocysteine levels and obstructive sleep apnea hypopnea syndrome: a systematic review and updated meta-analysis
Source: Front Endocrinol (Lausanne). 2024 Jun 3;15:1378293. doi: 10.3389/fendo.2024.1378293 (PMC11180825; doi:10.3389/fendo.2024.1378293)

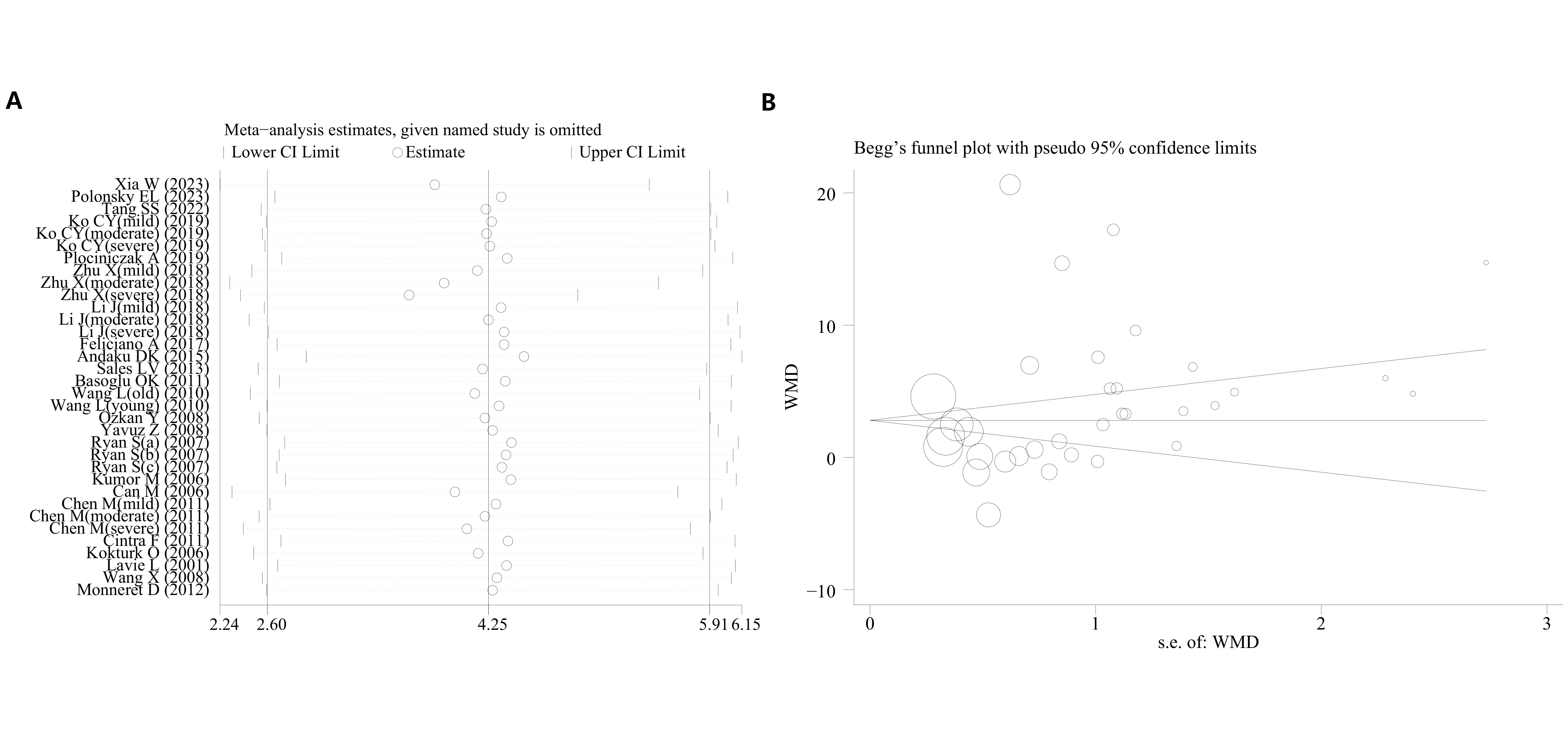

Supplement: Supplementary FIGURE 1 — Sensitivity analysis and funnel plots for the difference in HCY levels between controls and patients with OSAHS. (A) Sensitivity analysis, (B) Funnel plots of Begg’s test. [file Image_1.tif]

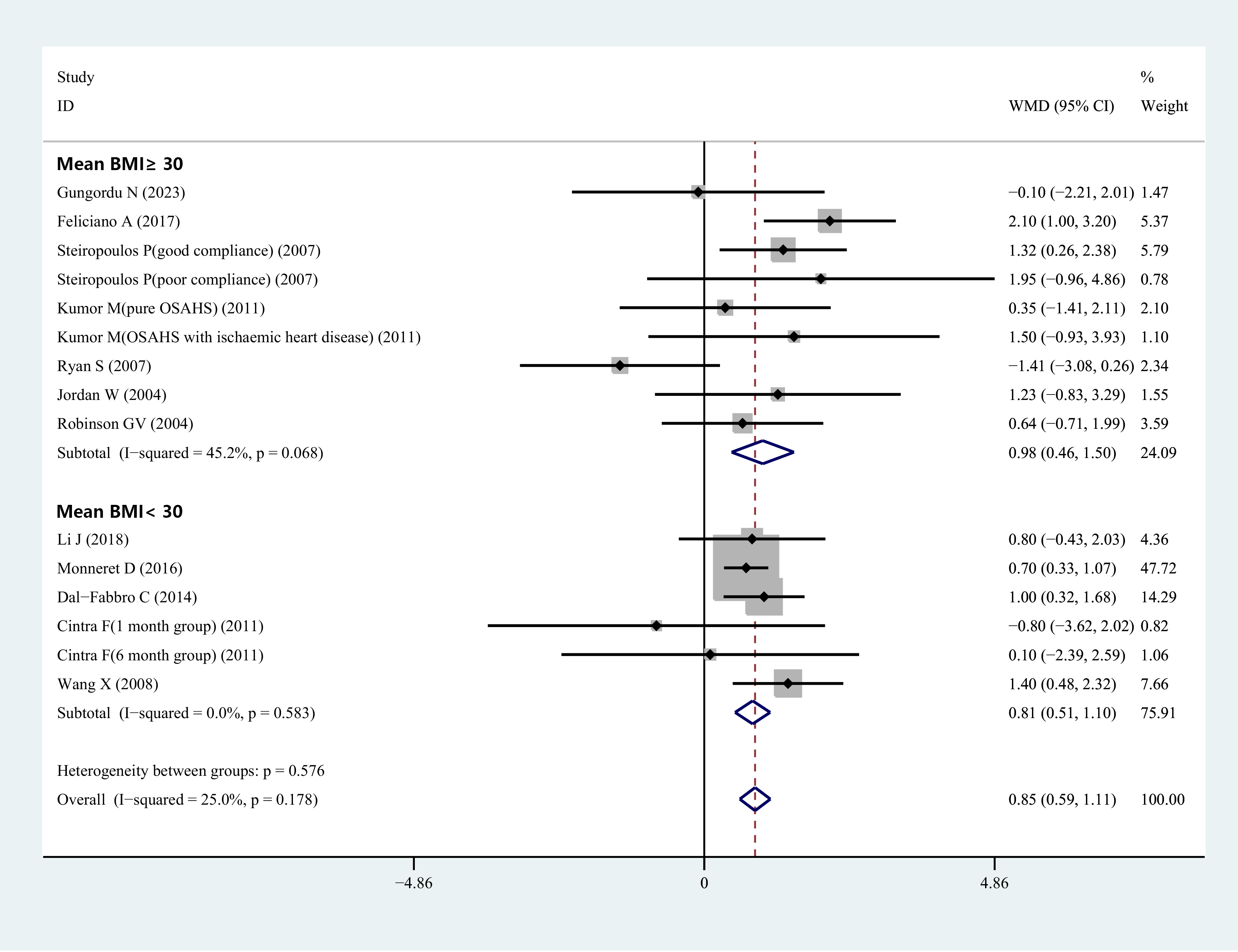

Supplement: Supplementary FIGURE 2 — Subgroup analysis depended on mean BMI of pre-CPAP treatment group compared to post-CPAP treatment group in meta-analysis. The means of expression are WMD and its 95%CI. [file Image_2.tif]

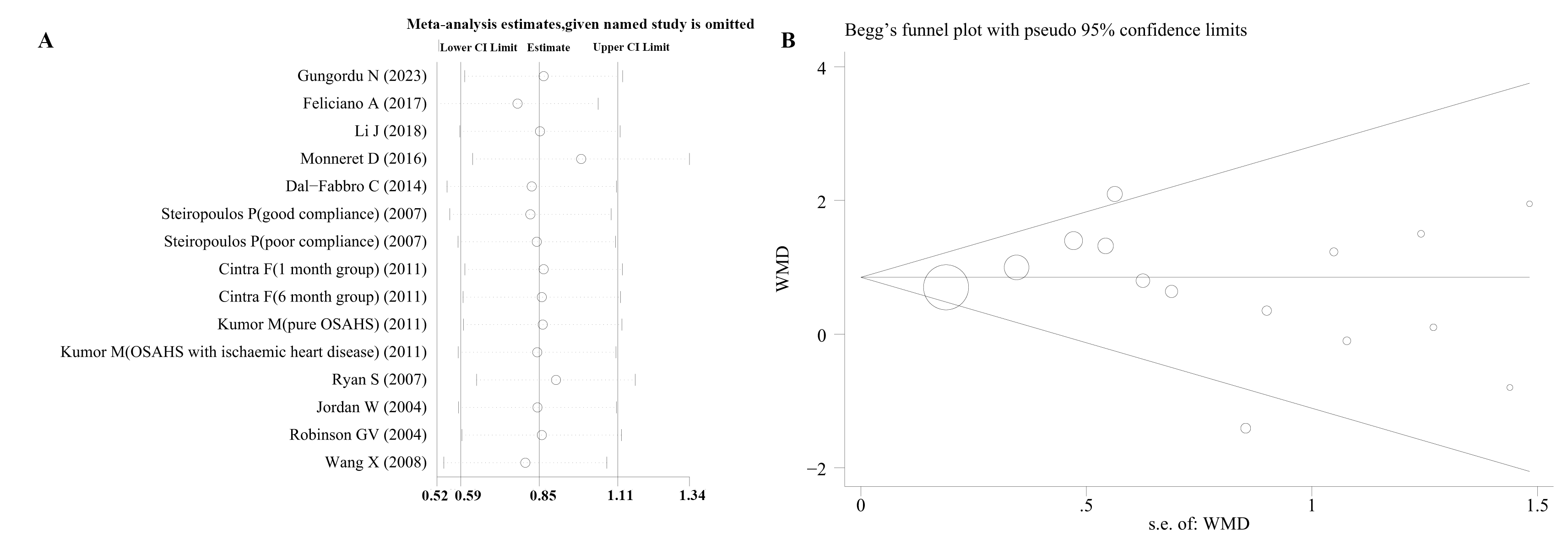

Supplement: Supplementary FIGURE 3 — Supplementary Figure 1 Sensitivity analysis and funnel plots for the difference in HCY levels between pre-CPAP treatment group and post-CPAP treatment group. (A) Sensitivity analysis, (B) Funnel plots of Begg’s test. [file Image_3.tif]
